# Supplementary material for: Accessing ultrastable glass via a bulk transformation
Source: Nat Commun. 2025 Jan 10;16:562. doi: 10.1038/s41467-024-55367-8 (PMC11724126; doi:10.1038/s41467-024-55367-8)
Supplement: Supplementary file 1 — Supplementary Information [file 41467_2024_55367_MOESM1_ESM.pdf]

Supplementary Information

Accessing ultrastable glass via a bulk transformation

Hengtong Bu[1], Hengwei Luan[1,2,3], Jingyi Kang[1], Jili Jia[1], Wenhui Guo[1], Yunshuai Su[1], Huaping Ding[4], Hsiang-Shun Chang[1], Ranbin Wang[1], You Wu[1], Lingxiang Shi[1], Pan Gong[4], Qiaoshi Zeng[5,6], Yang Shao[1*], Kefu Yao[1*]

[1] School of Materials Science and Engineering, Tsinghua University, Beijing 100084, China.

[2] Department of Mechanical Engineering, City University of Hong Kong, Hong Kong 999077, China.

[3] City University of Hong Kong Matter Science Research Institute (Futian), No. 3, Binglang Road, Futian District, Shenzhen 518045, China.

[4] State Key Laboratory of Materials Processing and Die & Mould Technology, School of Materials Science and Engineering, Huazhong University of Science and Technology, Wuhan 430074, China.

[5] Center for High Pressure Science and Technology Advanced Research, Shanghai 201203, China.

[6] Shanghai Key Laboratory of Material Frontiers Research in Extreme Environments (MFree), Institute for Shanghai Advanced Research in Physical Sciences (SHARPS), Shanghai 201203, China.

*: Corresponding authors

Yang Shao: shaoyang@tsinghua.edu.cn

Kefu Yao: kfyao@mail.tsinghua.edu.cn

Supplementary Figures

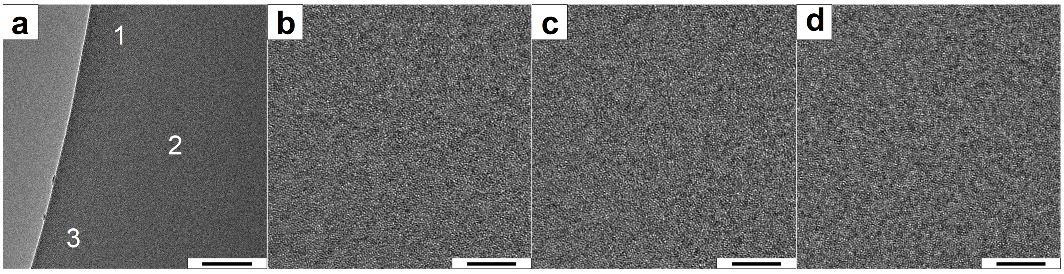

Supplementary Fig. 1 Additional TEM images of the heat-treated TiZrCuNiBe ribbons. a Bright-field TEM image (scale bar: 100 nm) of the heat-treated sample. **b** HRTEM (scale bar: 5 nm) image of area 1 in **a**. **c**, **d** Same as **b** but for areas 2 and 3 in **a**, respectively.

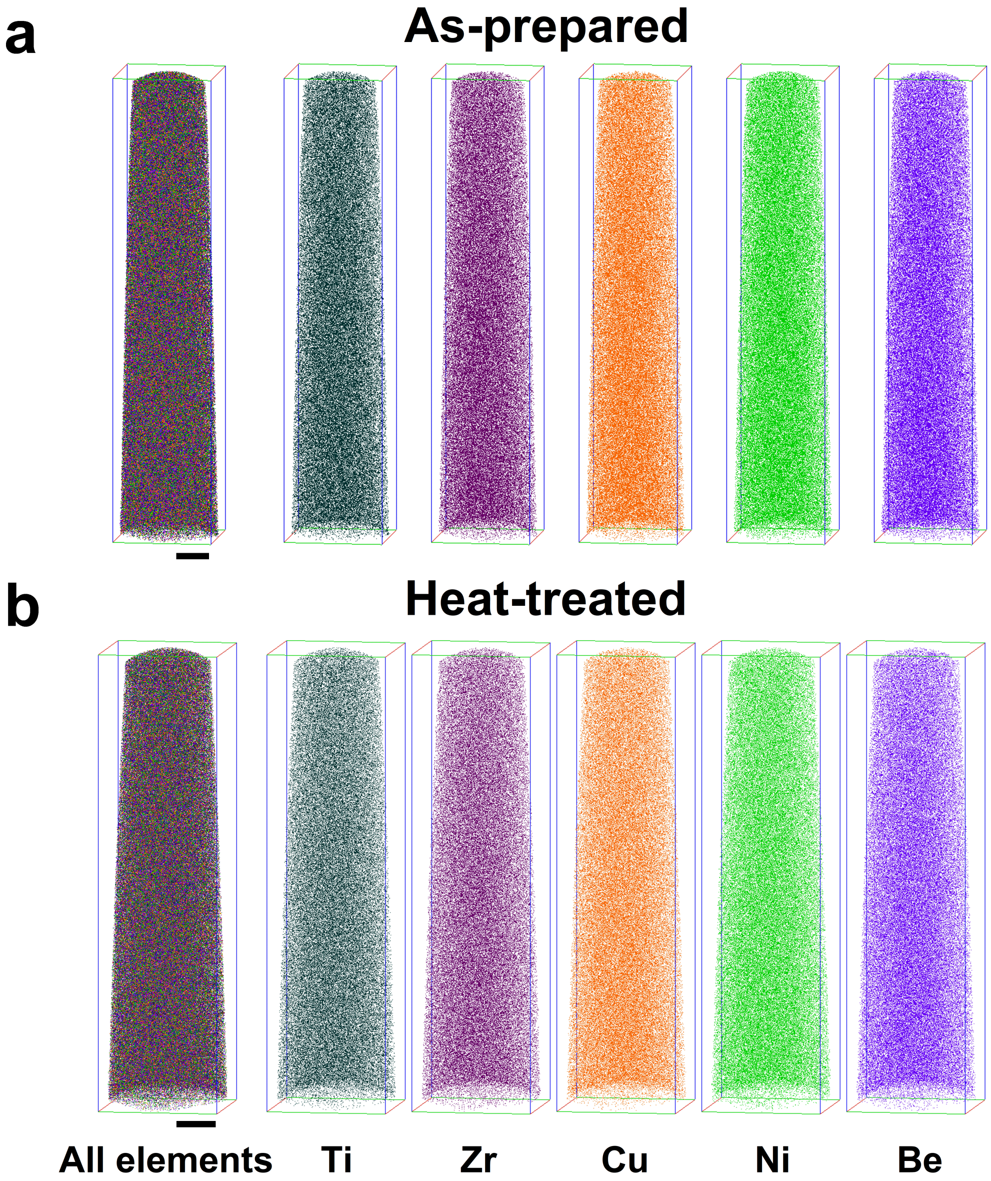

Supplementary Fig. 2 Atom probe tomography (APT) analyses of the as-prepared and heat-treated TiZrCuNiBe ribbons. a The APT results of the as-prepared sample. **b** The APT results of the heat-treated sample. Scale bar: 20 nm. The results show no compositional heterogeneity in sub-nanometer scales before and after the glass-to-glass transition.

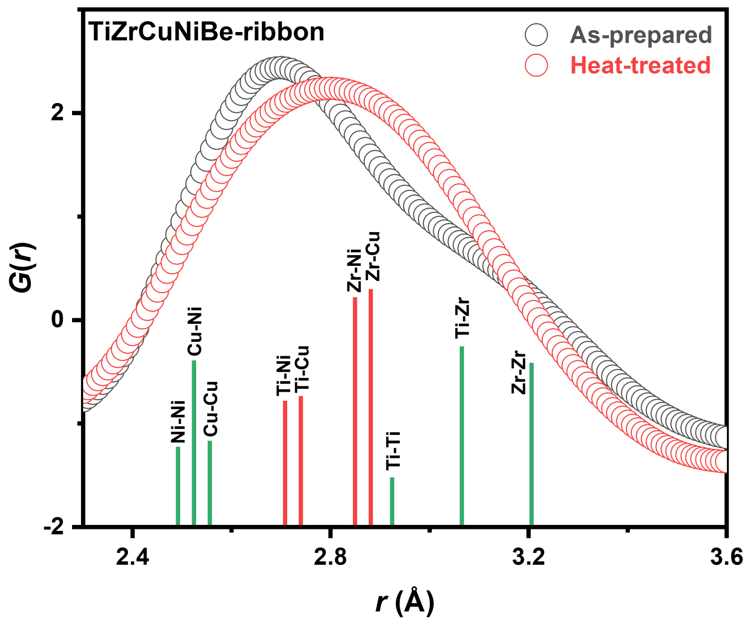

Supplementary Fig. 3 Analyses on the first peak of *G*(*r*) for the as-prepared and heat-treated TiZrCuNiBe ribbons. For a random quinary system, there are 15 atomic pairs contributed to the first peak of *G*(*r*). Considering the small atomic scattering factor of Be, the weights of Be-M (M refers to Ti, Zr, Cu, Ni, and Be) pairs are neglected. The other 10 atomic pairs are categorized into two groups and depicted in the figure: red bars represent atomic pairs with negative mixing enthalpy, while green bars represent atomic pairs with mixing enthalpy close to or equal to zero. Additionally, the height of each bar corresponds to the weight of each atomic pair. Qualitatively, after the glass-to-glass transition, there appears to be an increase in the proportion of atomic pairs with negative mixing enthalpy. Source data are provided as a Source Data file.

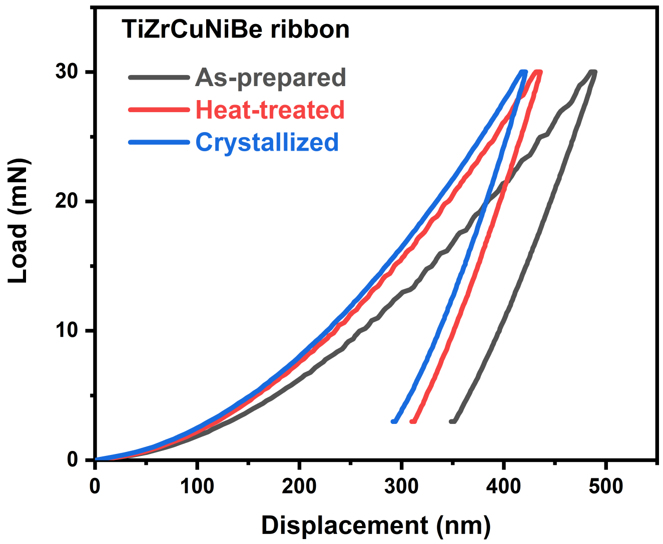

Supplementary Fig. 4 Typical load-displacement curves obtained by nanoindentation tests for the as-prepared, heat-treated, and crystallized TiZrCuNiBe ribbons. The hardness and reduced modulus are extracted and displayed in Table 1. Source data are provided as a Source Data file.

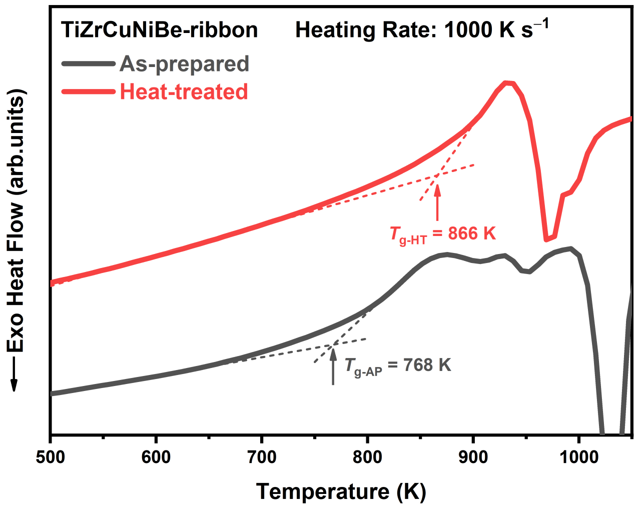

Supplementary Fig. 5 Typical flash DSC curves of the as-prepared and heat-treated TiZrCuNiBe ribbons. The heating rate was 1000 K s^{-1}. For each specimen, at least 3 curves were tested to exclude the possibility of macroscopic inhomogeneity and ensure accuracy. The glass transition temperatures are extracted and listed in Table 1 in the text. Source data are provided as a Source Data file.

Supplementary Notes

Supplementary Note 1. Discussion on the flash DSC curves.

The T_g of the as-prepared sample is covered by the relaxation process in the conventional DSC measurement with a heating rate of 10 K min^{-1}, as shown in Fig. 1a. To suppress the slow relaxation process and disclose the T_g, flash DSC measurements with a heating rate of 1000 K s^{-1} were conducted. The typical flash DSC curves are depicted in Supplementary Fig. 5, from which the T_gs of the as-prepared and heat-treated samples can be well detected. After multiple measurements, we confirmed the good repeatability of the test results, and the average values of T_gs for the as-prepared and heat-treated samples are listed in Table 1. After GGT, the T_g increased by 11.1%, indicating a significant enhancement in kinetic stability.

In addition, we also noticed a quite different crystallization process under flash heating. All the results exhibit good repeatability, eliminating the possibility of sample inhomogeneity. The explanation for these differences can be separated into two parts:

(1) For the as-prepared sample, compared to that under slow heating, the first and second post-T_g peaks become significantly weaker relative to the third exothermic peak under flash heating. This results from the effects of the crystallization kinetics. At a slow heating rate for the conventional DSC, all the processes, including structural relaxation, GGT, and the two-step crystallization, could fully develop. However, under flash heating, some processes would be suppressed, resulting in the vanished relaxation spectrum and the diminished first and second post-T_g peaks.

(2) Under flash heating, the crystallization processes of the as-prepared and heat-treated samples differ. The distinction can be attributed to the completely different energy states of the two samples. After GGT, the heat-treated sample has transformed into a lower energy state with a denser structure, whose crystallization dynamic could be expected different from that of the as-prepared one. Under slow heating, the two crystallization peaks of the as-prepared and heat-treated samples are quite similar as shown in Fig. 1, where the thermodynamic effects may dominate. However, under flash heating, the dynamic effects become prominent, resulting in different crystallization behavior. This, from another perspective, indicates different energy states and structural configurations of the two glasses.

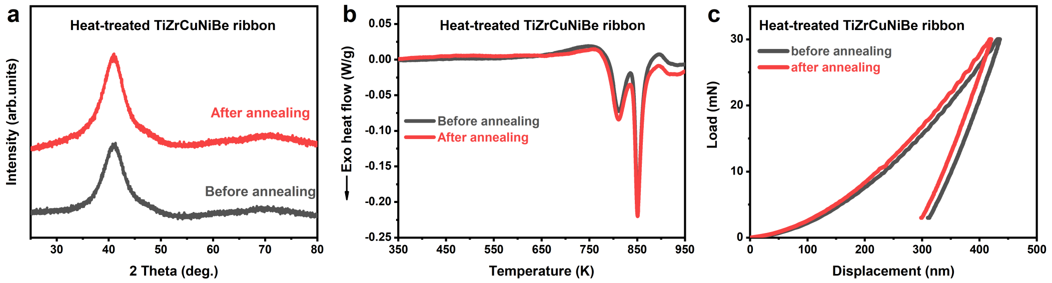

Supplementary Fig. 6 Excellent crystallization resistance and mechanical stability of the heat-treated TiZrCuNiBe glass. a XRD patterns and **b** DSC curves of the heat-treated sample before and after 10 h annealing at T_g + 70 K. No sharp Bragg peak was observed and the crystallization enthalpy showed no decline, indicating that the heat-treated sample remains amorphous after 10 h annealing. **c** Typical load-displacement curves obtained by nanoindentation tests for the heat-treated samples before and after 10 h annealing at T_g + 70 K. After 10 h annealing, the hardness and modulus increased only by 3.5% and 0.9%, respectively, manifesting good mechanical stability for the ultrastable TiZrCuNiBe glass. Source data are provided as a Source Data file.

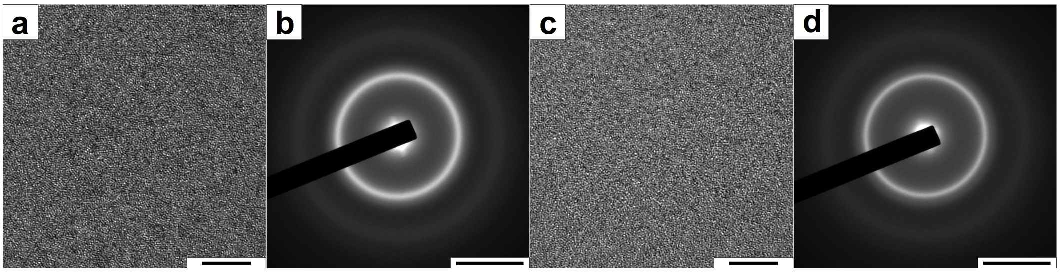

Supplementary Fig. 7 TEM analyses of the heat-treated $(TiZrCuNi)_{84}Be_{16}$ and $(TiZrCuNi)_{76}Be_{24}$ ribbons. **a** HRTEM image (scale bar: 5 nm) and **b** SAED image (scale bar: 5 nm^{-1}) of the heat-treated $(TiZrCuNi)_{84}Be_{16}$ sample. **c, d** Same as **a, b** but for the heat-treated $(TiZrCuNi)_{76}Be_{24}$ sample. No nanocrystals were observed in the two samples.

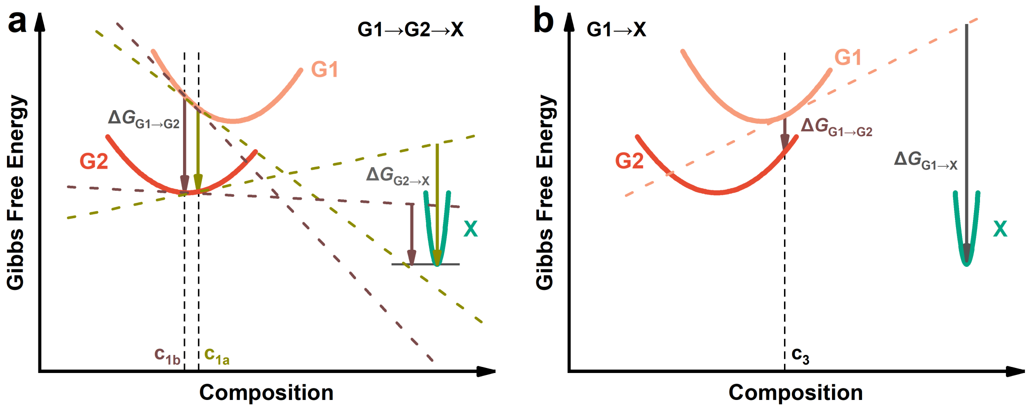

Supplementary Fig. 8 Additional illustrations on the competitive relationship between GGT and crystallization. a Influence of fine adjusting composition on the crystallization process of a well-separated GGT. When the composition point slightly shifts to the left (from c_{1a} to c_{1b}), the driving force of the precipitation of X from G2 becomes smaller, thereby leading to a suppressed crystallization. This can be used to explain the influence of Be content on the crystallization process for the B16, B20, and B24 glasses, which all exhibit a well-separated GGT. As the Be content increases (the composition point shifts to the left), the onset temperature of the second exothermic peak (corresponding to the crystallization event) increases (as seen in Fig. 4a), resulting from the decreased driving force of the precipitation of X from G2. **b** The positive driving force of the crystallization is much larger than that of GGT, in which case the crystallization dominates while the GGT is difficult to notice. We argue that most metallic glasses are probably under such circumstances.

Supplementary Tables

Supplementary Table 1. The peak position (Q_1) and integral width (W) of the FSDPs in $S(Q)$ for the as-prepared and heat-treated TiZrCuNiBe ribbons.

Sample	Q_1 (Å^{-1})	W (Å^{-1})
As-prepared	2.880 ± 0.001	0.549 ± 0.005
Heat-treated	2.891 ± 0.001	0.453 ± 0.004

Supplementary Table 2. Fitting results of the $G(r)$ peak heights versus peak positions using an exponential decay function $h(r)=A\exp(-r/\xi)$ for the as-prepared and heat-treated TiZrCuNiBe ribbons.

Sample	A (Å^{-2})	ξ (Å)	R^2
As-prepared	6.91 ± 0.31	2.59 ± 0.10	0.999
Heat-treated	4.31 ± 0.11	4.25 ± 0.11	0.999

Supplementary Table 3. Density increments caused by GGT, natural aging, and PVD.

Categories	Sample	Density increment (%)	Ref.
GGT	TiZrCuNiBe (this work)	2.30	this work
	$La_{32.5}Ce_{32.5}Co_{25}Al_{10}$	0.44	[1]
	$Pd_{42.5}Ni_{42.5}P_{15}$	0.61	[2]
Natural aging	110-million-year-aged amber	2.05	[3]
	17.7-year-aged $Ce_{70}Al_{10}Cu_{20}$	1.19	[4]
PVD	NHMe	1.30	[5]
	OMe	1.20	[5]
	Et	1.20	[5]
	DO37	1.30	[6]
	DSA-Ph	1.25	[7]
	TPD	1.35	[7]
	NPD	1.30	[8]
	EBZ	1.25	[9]
	CXB	1.10	[10]
	IMC	1.40	[11]
	α,α-A	1.36	[12]
	α,α-P	1.40	[13]
	β-AA	1.65	[13]
	α,α,α,α-TNBP	1.20	[13]
	TNB	1.43	[12]
	α,α,β-TNB	1.29	[14]
	PS	1.60	[15]

Supplementary Note 2. Additional discussion on density increments.

It is well-known that the density of a metallic glass is lower than that of its stable crystalline counterpart. Supplementary Table 4 summarizes the density differences ($\Delta\rho=(\rho_{crys}-\rho_{amor})/\rho_{amor}$) between some metallic glasses and their crystalline counterparts. The specific value of $\Delta\rho$ is influenced by the alloy composition and preparation process, but generally falls within the range of 1–4%. For glassy ribbons prepared by melt-spinning, it is widely accepted that their densities are 2–3% lower than those of crystalline counterparts[16]. In this work, the $\Delta\rho$ for the as-prepared and heat-treated samples are 3% and 0.7%, respectively. Compared to the $\Delta\rho$ values reported in the literature, the $\Delta\rho$ of the as-prepared sample falls in the typical range, while that of the heat-treated sample is rather small, further confirming the significant effect of the GGT in inducing a density increase. Given that relaxation typically results in a density increase of only 0.1–0.5%[17], the 2.3% density increase caused by GGT is so remarkable.

In Fig. 2b, the comparison of the density increments seems not quite fair due to the different cooling rates of the reference glasses for the deposited glasses and metallic glasses. The reference glasses are prepared by cooling the liquid at ~1 K min^{-1} for the deposited organic glass, but cooling at 10^2–10^6 K s^{-1} for metallic glasses. Bearing this in mind, we still made the comparison for two reasons:

(1) The glass-forming ability of most metallic glasses is quite limited, making it challenging to form them at cooling rates of a few K min^{-1}. Consequently, even in studies on the preparation of ultrastable metallic glasses by the PVD method[18,19], ribbon samples (i.e., metallic glasses obtained at a cooling rate of 10^4–10^6 K s^{-1}) are still used as references.

(2) The density of metallic glasses prepared at different cooling rates will vary, but the change is typically small. For example, the density difference of a $Pd_{40}Cu_{30}Ni_{10}P_{20}$ metallic glass prepared at cooling rate from 500 K s^{-1} to 1.98 K s^{-1} is only about 0.16%[20]. Furthermore, fitting the density data at different cooling rates of $Pd_{40}Cu_{30}Ni_{10}P_{20}$, results in an expression:

$$\rho = 0.0235 / R^{0.6551} + 9.2698 \quad (1)$$

with a coefficient of determination of 0.995, where ρ is the density and R is the cooling rate (R > 1.98 K s^{-1} to form a glass). Even if the cooling rate is very fast to approach infinity, the density would approach ρ_∞ = 9.2698 g cm^{-3}, which is only 0.164% difference from the glass with the cooling rate at 1.98 K s^{-1} (9.2850 g cm^{-3}). According to the fitting equation, density becomes even less sensitive to

the cooling rate, especially within the high cooling rate range.

For the above two reasons, we believe that the impact of the cooling rate accounts for only a small part of the increase in density, making the comparison still meaningful.

Supplementary Table 4. The density differences between metallic glasses and their crystalline counterparts.

	Compositions of metallic glasses (at.%)	$\Delta\rho$ (%)	Ref.
film	Cu-Zr	2.5–4.5	[21]
	Cu-Zr-Al	1–3	[22]
ribbon	$Cu_{33}Zr_{67}$	1.9	[23]
	$Cu_{59}Zr_{41}$	3.9	[23]
	Pd-Si	~2	[24]
	$Ti_{45}Zr_{5}Ni_{35}Cu_{15}$	2.11	[25]
	$Ti_{40}Zr_{10}Ni_{35}Cu_{15}$	1.74	[25]
	$Ti_{35}Zr_{15}Ni_{35}Cu_{15}$	1.26	[25]
	$Ti_{30}Zr_{20}Ni_{35}Cu_{15}$	1.08	[25]
bulk	$Ni_{59.5}Nb_{40.5}$	3.36	[26]
	Pd-Cu-Si	1.87–2.30	[27]
	Pd-Si-Cu	1.87–2.23	[27]
	Pd-Ni-Fe-P	0.62–1.34	[28]
	$Zr_{41.2}Ti_{13.8}Cu_{12.5}Ni_{10}Be_{22.5}$	1.09	[26]
	$Zr_{57}Cu_{15.4}Ni_{12.6}Al_{10}Nb_{5}$	2.32	[26]
	$Zr_{52.5}Cu_{17.9}Ni_{14.6}Al_{10}Ti_{5}$	2.23	[26]
	$Zr_{52}Ti_{5}Cu_{18}Ni_{15}Al_{10}$	2.58	[29]
	$Mg_{65}Cu_{7.5}Ni_{7.5}Zn_{5}Ag_{5}Y_{5}Gd_{5}$	0.98	[30]

Supplementary Table 5. δT_g and ΔT of some ultrastable metallic glasses.

Categories	Composition (at.%)	δT_g (%)	ΔT (K)	Ref.
GGT	TiZrCuNiBe	11.1 [a]	144	this work
	ZrTiHfCoNi	7.2 [a]	–	[31]
	$La_{32.5}Ce_{32.5}Co_{25}Al_{10}$	4.3 [b]	23	[1]
	$La_{65}Co_{25}Al_{10}$	11.1 [c]	83	[32]
	$Pd_{42.5}Ni_{42.5}P_{15}$	8.6 [c]<br>9.1 [b]	48	[2]
	$Pd_{32}Ni_{52}P_{16}$	4.7 [b]	112	[33]
Natural aging	17.7-year-aged $Ce_{70}Al_{10}Cu_{20}$	7.6 [b]	37	[4]
PVD	$Zr_{65}Cu_{27.5}Al_{7.5}$	1.6 [b]	40	[18]
	$Zr_{46}Cu_{46}Al_{8}$	8.5 [b]	44	[19]
	$Zr_{50}Cu_{41.5}Al_{5.5}Mo_{3}$	10.5 [b]	34	[34]
	$Zr_{55}Cu_{30}Ni_{5}Al_{10}$	7.1 [b]	193	[35]

Note: ΔTs were calculated using the data tested by conventional DSC at a heating rate of 10/20 K min^{-1}; δT_gs were calculated using the data tested by flash DSC at a heating rate of 1000/2000 K s^{-1} (marked by [a]), conventional DSC at a heating rate of 10/20 K min^{-1} (marked by [b]), or temperature-modulated DSC at a heating rate of 2/3 K min^{-1} (marked by [c]).

Supplementary References

1. Shen, J. *et al.* Metallic glacial glass formation by a first-order liquid-liquid transition. *J. Phys. Chem. Lett.* **11**, 6718-6723 (2020).
2. Du, Q. *et al.* Reentrant glass transition leading to ultrastable metallic glass. *Mater. Today* **34**, 66-77 (2020).
3. Perez-Castaneda, T., Jimenez-Rioboo, R. J. & Ramos, M. A. Two-level systems and boson peak remain stable in 110-million-year-old amber glass. *Phys. Rev. Lett.* **112**, 165901 (2014).
4. Zhao, Y. *et al.* Ultrastable metallic glass by room temperature aging. *Sci. Adv.* **8**, eabn3623 (2022).
5. Laventure, A., Gujral, A., Lebel, O., Pellerin, C. & Ediger, M. D. Influence of hydrogen bonding on the kinetic stability of vapor-deposited glasses of triazine derivatives. *J. Phys. Chem. B* **121**, 2350-2358 (2017).
6. Qiu, Y., Antony, L. W., de Pablo, J. J. & Ediger, M. D. Photostability can be significantly modulated by molecular packing in glasses. *J. Am. Chem. Soc.* **138**, 11282-11289 (2016).
7. Dalal, S. S., Walters, D. M., Lyubimov, I., de Pablo, J. J. & Ediger, M. D. Tunable molecular orientation and elevated thermal stability of vapor-deposited organic semiconductors. *PNAS* **112**, 4227-4232 (2015).
8. Esaki, Y., Komino, T., Matsushima, T. & Adachi, C. Enhanced electrical properties and air stability of amorphous organic thin films by engineering film density. *J. Phys. Chem. Lett.* **8**, 5891-5897 (2017).
9. Beasley, M. S., Bishop, C., Kasting, B. J. & Ediger, M. D. Vapor-deposited ethylbenzene glasses approach "ideal glass" density. *J. Phys. Chem. Lett.* **10**, 4069-4075 (2019).
10. Qiu, Y., Antony, L. W., Torkelson, J. M., de Pablo, J. J. & Ediger, M. D. Tenfold increase in the photostability of an azobenzene guest in vapor-deposited glass mixtures. *J. Chem. Phys.* **149**, 204503 (2018).
11. Dalal, S. S., Fakhraai, Z. & Ediger, M. D. High-throughput ellipsometric characterization of vapor-deposited indomethacin glasses. *J. Phys. Chem. B* **117**, 15415-15425 (2013).
12. Samanta, S. *et al.* Exploring the importance of surface diffusion in stability of vapor-deposited organic glasses. *J. Phys. Chem. B* **123**, 4108-4117 (2019).
13. Liu, T. *et al.* The effect of chemical structure on the stability of physical vapor deposited glasses of 1,3,5-triarylbenzene. *J. Chem. Phys.* **143**, 084506 (2015).
14. Dalal, S. S., Sepúlveda, A., Pribil, G. K., Fakhraai, Z. & Ediger, M. D. Density and birefringence of a highly stable α,α,β-trisnaphthylbenzene glass. *J. Chem. Phys.* **136** (2012).
15. Raegen, A. N., Yin, J., Zhou, Q. & Forrest, J. A. Ultrastable monodisperse polymer glass formed by physical vapour deposition. *Nat. Mater.* **19**, 1110-1113 (2020).
16. Suryanarayana, C. & Inoue, A. Bulk metallic glasses.
17. Goncharova, E. V., Konchakov, R. A., Makarov, A. S., Kobelev, N. P. & Khonik, V. A. On the nature of density changes upon structural relaxation and crystallization of metallic glasses. *J. Non-Cryst. Solids* **471**, 396-399 (2017).
18. Yu, H. B., Luo, Y. & Samwer, K. Ultrastable metallic glass. *Adv. Mater.* **25**, 5904-5908 (2013).
19. Luo, P. *et al.* Ultrastable metallic glasses formed on cold substrates. *Nat. Commun.* **9**, 1389 (2018).
20. Hu, X., Ng, S. C., Feng, Y. P. & Li, Y. Cooling-rate dependence of the density of $Pd_{40}Ni_{10}Cu_{30}P_{20}$ bulk metallic glass. *Physical Review B* **64** (2001).
21. Li, Y., Guo, Q., Kalb, J. A. & Thompson, C. V. Matching glass-forming ability with the density of the amorphous phase. *Science* **322**, 1816-1819 (2008).
22. Guo, Q. *et al.* Density change upon crystallization of amorphous Zr-Cu-Al thin films. *Acta Mater.* **58**, 3633-3641 (2010).
23. Altounian, Z., Guo-hua, T. & Strom-Olsen, J. O. Crystallization characteristics of Cu-Zr metallic glasses from $Cu_{70}Zr_{30}$ to $Cu_{25}Zr_{75}$. *J. Appl. Phys.* **53**, 4755-4760 (1982).
24. Masumoto, T., Kimura, H., Inoue, A. & Waseda, Y. Structural stability of amorphous metals. *Materials Science and Engineering* **23**, 141-144 (1976).

25. Kim, W.-C. *et al.* Enhancement of superelastic property in Ti-Zr-Ni-Cu alloy by using glass alloy precursor with high glass forming ability. *Acta Mater.* **173**, 130-141 (2019).
26. Mukherjee, S., Schroers, J., Zhou, Z., Johnson, W. L. & Rhim, W. K. Viscosity and specific volume of bulk metallic glass-forming alloys and their correlation with glass forming ability. *Acta Mater.* **52**, 3689-3695 (2004).
27. Chen, H. S. & Park, B. K. Role of chemical bonding in metallic glasses. *Acta Metall.* **21**, 395-400 (1973).
28. Shen, T. D., He, Y. & Schwarz, R. B. Bulk amorphous Pd-Ni-Fe-P alloys: Preparation and characterization. *J. Mater. Res.* **14**, 2107-2115 (1999).
29. Mattern, N. *et al.* Thermal behavior and glass transition of Zr-based bulk metallic glasses. *Materials Science and Engineering: A* **375-377**, 351-354 (2004).
30. Park, E. S. & Kim, D. H. Formation of Mg–Cu–Ni–Ag–Zn–Y–Gd bulk glassy alloy by casting into cone-shaped copper mold in air atmosphere. *J. Mater. Res.* **20**, 1465-1469 (2005).
31. Yang, Q., Yang, X.-M., Zhang, T., Liu, X.-W. & Yu, H.-B. Structure and entropy control of polyamorphous transition in high-entropy metallic glasses. *Acta Mater.* **266** (2024).
32. Yang, Q. *et al.* Structural length-scale of beta relaxation in metallic glass. *J. Chem. Phys.* **157**, 184504 (2022).
33. Ouyang, L. F. *et al.* Strong-to-fragile transition in a metallic-glass forming supercooled liquid associated with a liquid–liquid transition. *J. Appl. Phys.* **133** (2023).
34. Sun, Q. *et al.* Transition towards ultrastable metallic glasses in Zr-based thin films. *Appl. Surf. Sci.* **533** (2020).
35. Aji, D. P. B. *et al.* Ultrastrong and ultrastable metallic glass. Preprint at https://arxiv.org/abs/1306.1575 (2013).
